# Key features of pneumococcal isolates recovered in Central and Northwestern Russia in 2011–2018 determined through whole-genome sequencing

**DOI:** 10.1099/mgen.0.000851

**Published:** 2022-09-16

**Authors:** Ekaterina Egorova, Narender Kumar, Rebecca A. Gladstone, Yulia Urban, Elena Voropaeva, A.V. Chaplin, Elena Rumiantseva, T. S. Svistunova, Paulina A. Hawkins, Keith P. Klugman, Robert F. Breiman, Lesley McGee, Stephen D. Bentley, Stephanie W. Lo

**Affiliations:** ^1^​ G. N. Gabrichevsky Research Institute for Epidemiology and Microbiology, Moscow, Russia; ^2^​ Parasites and Microbes, Wellcome Sanger Institute, Hinxton, UK; ^3^​ Department of Biostatistics, Institute of Basic Medical Sciences, Faculty of Medicine, University of Oslo, Oslo, Norway; ^4^​ Labstory Medical Center, Saint Petersburg, Russia; ^5^​ Infectious Clinical Hospital № 2, Moscow, Russia; ^6^​ Centers for Disease Control and Prevention, Atlanta, USA; ^7^​ Rollins School of Public Health, Emory University, Atlanta, Georgia, USA; ^8^​ Emory Global Health Institute, Atlanta, USA; ^9^​ Department of Pathology, University of Cambridge, Cambridge, UK

**Keywords:** antimicrobial resistance, global pneumococcal sequence cluster (GPSC), Russia, serotypes, *Streptococcus pneumoniae*, WGS (whole-genome sequencing)

## Abstract

Invasive pneumococcal disease remains one of the leading causes of morbidity and mortality worldwide. In Russia, 13- valent pneumococcal conjugate vaccine (PCV13) was introduced into the childhood immunization programme nationwide in 2014. As part of the Global Pneumococcal Sequencing Project (GPS), we used genome data to characterize 179 pneumococcal isolates collected from Russia in 2011–2018 to investigate the circulating pneumococcal strains using a standardized genomic definition of pneumococcal lineages (global pneumococcal sequence clusters, GPSCs), prevalent serotypes and antimicrobial resistance profiles.

We observed high serotype and lineage diversity among the 179 isolates recovered from cerebrospinal fluid (*n*=77), nasopharyngeal swabs (*n*=99) and other non-sterile site swabs (*n*=3). Overall, 60 GPSCs were identified, including 48 clonal complexes (CCs) and 14 singletons, and expressed 42 serotypes (including non-typable). Among PCV13 serotypes, 19F, 6B and 23F were the top three serotypes while 11A, 15B/C and 8 were the top three among non-PCV13 serotypes in the collection. Two lineages (GPSC6 and GPSC47) expressed both PCV13 and non-PCV13 serotypes that caused invasive disease, and were penicillin- and multidrug-resistant (MDR), highlighting their potential to adapt and continue to cause infections under vaccine and antibiotic selective pressure. PCV13 serotypes comprised 92 % (11/12) of the CSF isolates from the children aged below 5 years; however, the prevalence of PCV13 serotype isolates dropped to 53 % (31/58) among the nasopharyngeal isolates. Our analysis showed that 59 % (105/179) of the isolates were predicted to be non-susceptible to at least one class of antibiotics and 26 % (46/179) were MDR. Four MDR lineages (GPSC1, GPSC6, GPSC10 and GPSC47) accounted for 65 % (30/46) of the MDR isolates and expressed PCV13 serotypes (93 %, 28/30).

This study provides evidence of high genetic and serotype diversity contributed by a mix of globally spreading and regionally circulating lineages in Russia. The observations suggest that the PCV13 vaccine could be important in reducing both invasive disease and antimicrobial resistance. We also identify potential lineages (GPSC6 and GPSC47) that may evade the vaccine.

## Data Summary

Genome sequences were deposited in the European Nucleotide Archive (ENA) and the accession numbers are available in the metadata. A phylogenetic snapshot is available at https://microreact.org/project/GPS_Russia. The authors confirm all supporting data, code and protocols have been provided within the article or through supplementary data files.

**Impact Statement**

This study provides a comprehensive characterization of pneumococcal isolates collected in Russia based on whole-genome sequencing (WGS). In this work, we analysed genomic data from *Streptococcus pneumoniae* isolates recovered from individuals with meningitis, acute upper respiratory tract infection and non-invasive disease (conjunctivitis and bartholinitis) in Moscow, Central and Northwestern Federal Districts between 2011 and 2018 to provide a detailed description of key features of the collection in an international context. We revealed high serotype and lineage diversity in the collection. We identified that the dominant pneumococcal vaccine serotypes (VTs) were associated with globally spreading lineages (GPSC6 and GPSC47) that also include non-vaccine serotypes (NVTs), indicating that they can potentially contribute to vaccine evasion. Overall, this study represents a significant contribution to our understanding of pneumococcal epidemiology in Russia within a global context.

## Introduction

*Streptococcus pneumoniae* is an opportunistic pathogen that can cause mild infections such as otitis media as well as life-threatening invasive pneumococcal disease (IPD) (e.g. meningitis and sepsis). It is one of the leading causes of mortality and morbidity among children worldwide [1, 2]. In 2008, pneumococcal infections were responsible for 476 000 deaths among HIV-negative children under 5 years of age globally [3]. By 2015, pneumococcal death was reduced to 294 000 in the same age group; such a decline is considered to be a result of the introduction of pneumococcal conjugate vaccines (PCVs) in regions with high child mortality [4]. Vaccination is regarded as the most effective measure for IPD prevention, and the World Health Organization (WHO) recommends that PCVs are included in routine childhood immunization programmes in all countries [4, 5]. The first seven-valent pneumococcal conjugate vaccine (PCV7) was introduced in 2000 and contained capsule polysaccharides of seven serotypes (14, 6B, 19F, 23F, 4, 9V, 18C). Subsequently, PCV10 (PCV7 serotypes plus 1, 5, 7F) and PCV13 (PCV10 serotypes plus 3, 6A, 19A) were licensed in 2009 and 2010, respectively. Despite the success of PCVs, IPD remains an important health priority owing to an increase in disease caused by pneumococci expressing non-vaccine serotypes (NVTs) and persistent circulation of certain vaccine serotypes (VTs) [6–13].

In Russia, PCV13 was introduced into the childhood immunization programme nationwide as a three-dose schedule at 2, 4.5 and 15 months of age in 2014 [14]. According to the WHO and United Nations Children's Fund (UNICEF) estimates of vaccination coverage, PCV13 uptake in Russia increased from 35 % in 2016 to over 80 % in 2019 [15]. A study by Briko *et al*. [16] reported that 88 % of children aged over 12 months had received their first dose of vaccine by 2017. In the same study, PCV13 vaccine uptake measured by the first dose was 62 % in Moscow Oblast in 2017 and 74 % in Leningrad Oblast (in the Northwestern Federal District) in 2016 [16]. It was also noted that the uptake rates varied throughout the country, ranging from 23 % in Chechen Republic to 99 % in Krasnodar Krai [16].

There is currently no national surveillance system for pneumococcal disease in Russia [17, 18]. However, a number of studies have reported data on pneumococcal infection incidence and distribution of circulating pneumococcal serotypes [18–23]. *S. pneumoniae* was responsible for 2–20 % of bacterial meningitis cases in children under 5 years of age in 2005–2006, and the incidence of pneumococcal meningitis in the same age group was 4–8 cases per 100 000 [17, 19]. Before PCV13 introduction, it was estimated that the vaccine would cover 66.2–90.4 % of circulating pneumococcal serotypes, depending on the patients’ age group, geographical region and form of clinical manifestation. PCV13 serotypes 6B, 14, 19F, 23F and 3 were found to be the most prevalent in the country [19–22]. A previous study in Moscow showed a significant decline of PCV13 serotypes from 77.7 % in 2015 to 58.5 % in 2017 among nasopharyngeal isolates collected from children with upper respiratory infections aged less than 5 years after vaccine introduction; however, an increase was noted in NVTs, mainly due to increased prevalence of serotype 15B/C isolates associated with ST1025 and ST1262 [23]. A multicentre carriage study among healthy children aged 6 years and under across six cities in Russia in 2016–2018 showed an overall decrease in the prevalence of PCV13 serotypes (VTs) between vaccinated and unvaccinated groups. Additionally, increased prevalence of non-vaccine serotypes, particularly 15 A/F, was reported among vaccinated children compared with unvaccinated children [18].

Although whole-genome sequencing (WGS) has been increasingly used in pathogen surveillance globally, most previous studies from Russia reported the prevalent serotypes and their associated multi-locus sequence types (MLSTs) using traditional methods [20–23]. There is only one study that applied WGS to characterize circulating pneumococcal strains during the introduction of PCV13 in Russia [24]. From the sequencing of 46 IPD isolates, Mironov *et al*. identified 23 serotypes, of which the predominant serotypes 3 and 19F were expressed by 5 sequence types (STs) (ST180, 505, 2049, 15250, 15251) and 4 STs (ST15, 179, 230, 236), respectively [24].

In this study, we analysed the genome sequences of 179 pneumococcal isolates collected from Central and Northwestern Russia between 2011 and 2018. We investigated key features of the pneumococcal isolates, including pneumococcal lineages or global pneumococcal sequence clusters (GPSCs) [25], *in silico* serotype, MLST and antimicrobial resistance. These characteristics were then analysed in conjunction with the clinical metadata to gain insights into the serotype and lineage diversity and antimicrobial resistance in the collection.

## Methods

### Bacterial collection

In this study, the pneumococcal collection included isolates from invasive disease [cerebrospinal fluid (CSF), *n*=77], from nasopharyngeal swabs (NPSs) from individuals with acute upper respiratory infections (*n*=99) and from non-invasive pneumococcal diseases (*n*=3) Table S1 (available in the online version of this article).

CSF samples were collected from patients diagnosed with meningitis at Moscow Clinical Infectious Hospital #2 between 2011 and 2018. It is the largest infectious diseases hospital with a capacity of 810 beds. On average 20 000 patients are admitted to the hospital each year, including residents of both Moscow and other regions of the country.

NPSs from individuals with acute upper respiratory infections were collected as part of a routine diagnostic procedure at two facilities. First, Moscow G. N Gabrichevsky Research Institute for Epidemiology and Microbiology (MGRIEM) Diagnostic Center between 2011–2018. In this period, MGRIEM served 70 884 patients, who were mainly residents of Moscow. Second, the Labstory Medical Center between March and June in 2018, which served 11 082 patients, mainly from Northwestern Federal District of Russia, in this period. Overall, a total of 23 664 and 3692 NPSs were collected from these 2 facilities, respectively. A total of 570 pneumococcal isolates were initially recovered from 27 356 nasopharyngeal swabs. Ninety-nine nasopharyngeal isolates included in this study were selected randomly from among them.

Pneumococcal isolates from non-invasive disease were collected from the Labstory Medical Center between 2011–2018. They were isolated from eye swabs (*n*=2) from patients diagnosed with conjunctivitis and a vaginal swab (*n*=1) from an individual with bartholinitis.

### Species identification and antimicrobial susceptibility testing

All clinical samples were collected by routine methods and later inoculated on Columbia agar plates (Oxoid, UK) supplemented with 5 % sheep blood. Plates were incubated at 37 °C in 5 % CO_2_ for 18–24 h. Pneumococcal isolates were identified by the optochin sensitivity test (Oxoid, UK), the bile solubility test and real-time PCR specific for *lytA* [26]. We serotyped the isolates by latex agglutination and quellung reaction with the sets of pooled and individual antisera (SSI, Denmark) [27, 28]. Subsequently, all the isolates were stored at −80 °C in 10 % skim milk and 15 % glycerol solution.

Antibiotic susceptibility testing against penicillin (PEN), cefotaxime (CTX), erythromycin (ERY), sulfamethoxazole/trimethoprim (SXT), chloramphenicol (CAT) and tetracycline (TET) was conducted by disc diffusion method, E-tests, broth microdilutions or agar dilutions using Mueller–Hinton agar (Oxoid, UK) supplemented with 5 % sheep blood. *S. pneumoniae* ATCC 49619 was used as the control reference strain. The results were interpreted using Clinical and Laboratory Standards Institute (CLSI) guidelines (M100-ED28 : 2018) [29]. Penicillin resistance was defined as an MIC of ≥0.12 µg ml^−1^, according to meningitis breakpoints.

### Genome sequencing and data analyses

Isolates were sequenced as part of the Global Pneumococcal Sequencing (GPS) Project (http://www.pneumogen.net/gps/). Briefly, genomic DNA was extracted from the pneumococcal isolates using a modified QIAamp DNA mini kit protocol (Qiagen, Inc., Valencia, CA, USA) as previously described [30]. The pneumococcal isolates were whole-genome sequenced on an Illumina HiSeq platform to produce paired-end reads of 151 bp in length and raw data were deposited in the European Nucleotide Archive (ENA) (Metadata S1). WGS data were processed as previously described [25, 30]. In brief, the serotypes and MLST were derived from genome data using SeroBA [31] and MLSTcheck [32], respectively. The predicted resistance against beta-lactam antibiotics, erythromycin, clindamycin, quinupristin/dalfopristin, linezolid, cotrimoxazole, tetracycline, levofloxacin, chloramphenicol, rifampin and vancomycin was derived from the genomic data [33–35]. Among these, five commonly used antibiotics – penicillin, chloramphenicol, cotrimoxazole, erythromycin and tetracycline – were used for resistance pattern analysis and other predictions were recorded in Metadata S1. All the isolates were classified as susceptible, intermediate or resistant according to CLSI guidelines (M100-ED28 : 2018) [29]. Penicillin resistance was defined as an MIC of ≥0.12 µg ml^−1^, according to meningitis breakpoints. Multidrug resistance was defined as resistance to three or more classes of antibiotics.

Each isolate was assigned to a GPSC using PopPUNK and a reference list of pneumococcal isolates (*n*=42 160) (https://www.pneumogen.net/gps/assigningGPSCs.html) [25, 36]. Using the GPS database (last accessed 8 March 2021, *n*=26 100) that also included isolates sequenced in this study, we classified the GPSCs into dominant GPSCs (represented by >100 isolates), intermediate GPSCs (10–100) and rare GPSCs (<10) [25]. Local GPSCs were lineages identified only in Russia and not found elsewhere based on the GPS database and MLST database (https://pubmlst.org/spneumoniae/, last accessed March 2021). Clonal complex (CC) was assigned using the single locus variant (SLV) threshold on the GPS dataset as previously described [25]. Phylogenetic analysis was performed on all isolates by constructing a maximum-likelihood tree using FastTree v2.1.10, based on single-nucleotide polymorphisms (SNPs) extracted from an alignment generated by mapping reads to the reference genome of *S. pneumoniae* ATCC 700 669 [National Center for Biotechnology Information (NCBI) accession number FM211 187]. To place the GPSCs of interest in global context, GPSC/lineage-specific global phylogenies were generated using isolates from Russia and other countries in the GPS database. For each GPSC, reads from each sample were mapped to a GPSC-specific reference genome [37] to create an alignment using Burrows–Wheeler Aligner v0.7.17-r1188 (BWA). Recombination regions were then removed using Gubbins v2.4.1 [38]. Recombination-free alignment was then reduced to SNP alignment to create a maximum-likelihood tree using RAxMLv8.2.8 [39]. Capsular or serotype switching was detected when isolates had identical STs but expressed different serotypes. The metadata and analysis results can be visualized interactively online using the Microreact tool at https://microreact.org/project/GPS_Russia.

### Statistical analysis

The isolates were categorized as VT if the serotype predicted was included in the PCV13 vaccine and NVT if it was not. Prevalence between categories was compared using Fisher’s exact test and multiple testing was adjusted using the Benjamini–Hochberg false discovery rate of 5 %. Prior to carrying out Fisher’s exact test, a power calculation using R package pwr was used to estimate the sample size to achieve 80 % statistical power with a significance level of *P* value <0.05 [40]. If the number of samples was below the sample size from the power calculation, Fisher’s exact test was not carried out. Two-sided *P* values of <0.05 were considered statistically significant. The statistical analysis was carried out in R version 3.5.2.

## Results

### Circulating pneumococcal lineages

The pneumococcal collection in this study showed high genetic diversity with 102 known and 11 novel STs (represented solely by isolates from Russia) identified among 179 isolates. The 113 STs were grouped into 48 CCs and 14 ST singletons. The CC assignments were in high concordance with the GPSCs (Fig. S1). Of the 60 GPSCs identified, the 5 most frequent GPSCs that formed 31 % (56/179) of the entire collection were: GPSC1 (CC320, *n*=12) expressing serotypes 19A/19F, GPSC3 (CC53/62/1012, *n*=12) expressing serotypes 8/11A, GPSC7 (CC439, *n*=11) expressing serotypes 23A/23F, GPSC47 (CC386, *n*=11) expressing serotypes 6B/6C and GPSC12 (CC180, *n*=10) expressing serotype 3 (Tables 1 and S2). In order to gain further understanding of the genetic relatedness of the Russian isolates with global isolates in the GPS database, we constructed global lineage-specific phylogenetic trees of the top five GPSCs (GPSC1, GPSC3, GPSC7, GPSC47 and GPSC12) (Figs S2, S3 and S4). We did not observe any close clustering of Russian isolates to indicate any country-specific cluster except for GPSC12, where 8 of the 10 isolates were grouped within a cluster Fig. S4A, B. The majority (21/24) of the isolates within this cluster belonged to the Russian Federation and neighbouring Eastern European countries, showing a close genetic relatedness among them. The analysis of the lineage-specific trees therefore suggests multiple importations of these lineages into the Russian Federation. However, the limited number of samples in the current study did not allow us to further identify whether these lineages were established and circulating within the Russian Federation via clonal expansion.

**Table 1.** GPSCs identified with associated clonal complexes, sequence types and serotypes (extract from Table S2)

GPSC	CC or ST (related PMEN clone*)	No. of isolates	CSF†	NPS‡, eye or vaginal swabs	*In silico* serotype (*n*)
1	CC320 (Taiwan^19F^-14/DLV)	12	6	6	**19F (11), 19A (1)**
3	CC62 (Netherlands ^8^-33/SLV)	12	1	3	11A (4)
	CC1012		2	4	11A (6)
	CC53 (Netherlands ^8^-33/SLV)		2	0	8 (2)
7	CC439 (Tennessee^23F^-4/SLV)	11	4	7	**23F (8),** 23A (3)
47	CC386	11	3	8	6B (9), 6C (2)
12	CC180 (Netherlands^3-^31/DLV)	10	2	8	**3** (**10**)
6	CC156 (Spain^9V^-3/SLV)	8	2	6	**19F (1)**, 14 (4), 15A (2), 11A (1)
44	CC177 (Portugal^19F^-21/SLV)	7	2	5	**19F (7)**
162	CC1222 Poland^6B^-20/DLV)	7	7	0	**4** (**7**)
11	CC1262	6	1	3	15B/C (3), **19F (1)**
	CC193(Greece^21^ −30/DLV)		0	2	15A (2)
19	CC433	5	1	4	22F (4),42 (1)
32	CC218 (Denmark^12F^-24/SLV)	5	4	1	**7F (2)**, 8 (3)
Other GPSCs§ *n*=49	Other CCs (*n*=34) and ST singletons (*n*=14)	85	40	45	**1 (1)**, 10A (3), 10B (1), 10F (1), 12F (3), 14 (3), 15F (1), 15B/C (7), 16F (1), 17F (1), 18A (1), **18C (4)**, 18F(1), **19A (1)**, **19F (6)**, 20B (1), 22F (1), 23A (2), 23B (1), **23F (6)**, 25F (2), 28A (2), 34 (2), 35B (1), 35F (3), 36 (3), 37 (1), **5 (1)**, **6A (7)**, 6B (7), 6C (1), **7F (1)**, **9V (2)**, 9L (1), 9N (3), NT (2)
Total	48 CCs and 14 singletons	179	77	102	179

PCV13 serotypes are highlighted in bold.

SLV, single-locus variant.

DLV, double-locus variant.

*PMEN website: http://web1.sph.emory.edu/PMEN/pmen_table2.html

†CSF, cerebrospinal fluid.

‡NPS, nasopharyngeal swabs.

§Other GPSCs represented by fewer than five isolates.

PMEN, Pneumococcal Molecular Epidemiology Network

Of the 60 GPSCs, 25 were classified as dominant GPSCs represented by 66 % (118/179), 19 intermediate GPSCs represented by 24 % (43/179) and 16 rare GPSCs represented by 10 % (18/179) of the collection. Among the rare GPSCs, there were 10 GPSCs (GPSC310, GPSC390, GPSC566-70, GPSC572, GPSC591 and GPSC629) mainly found in Eastern Europe and West Asia Tables S3 and S4, suggesting that they could be regionally circulating lineages. Notably, of the seven globally spreading GPSCs reported by Gladstone *et al*. [25], five (GPSC1, GPSC6, GPSC7, GPSC18 and GPSC23) were also found in this study.

### Prevalent serotypes and associated lineages

**Fig. 1.**
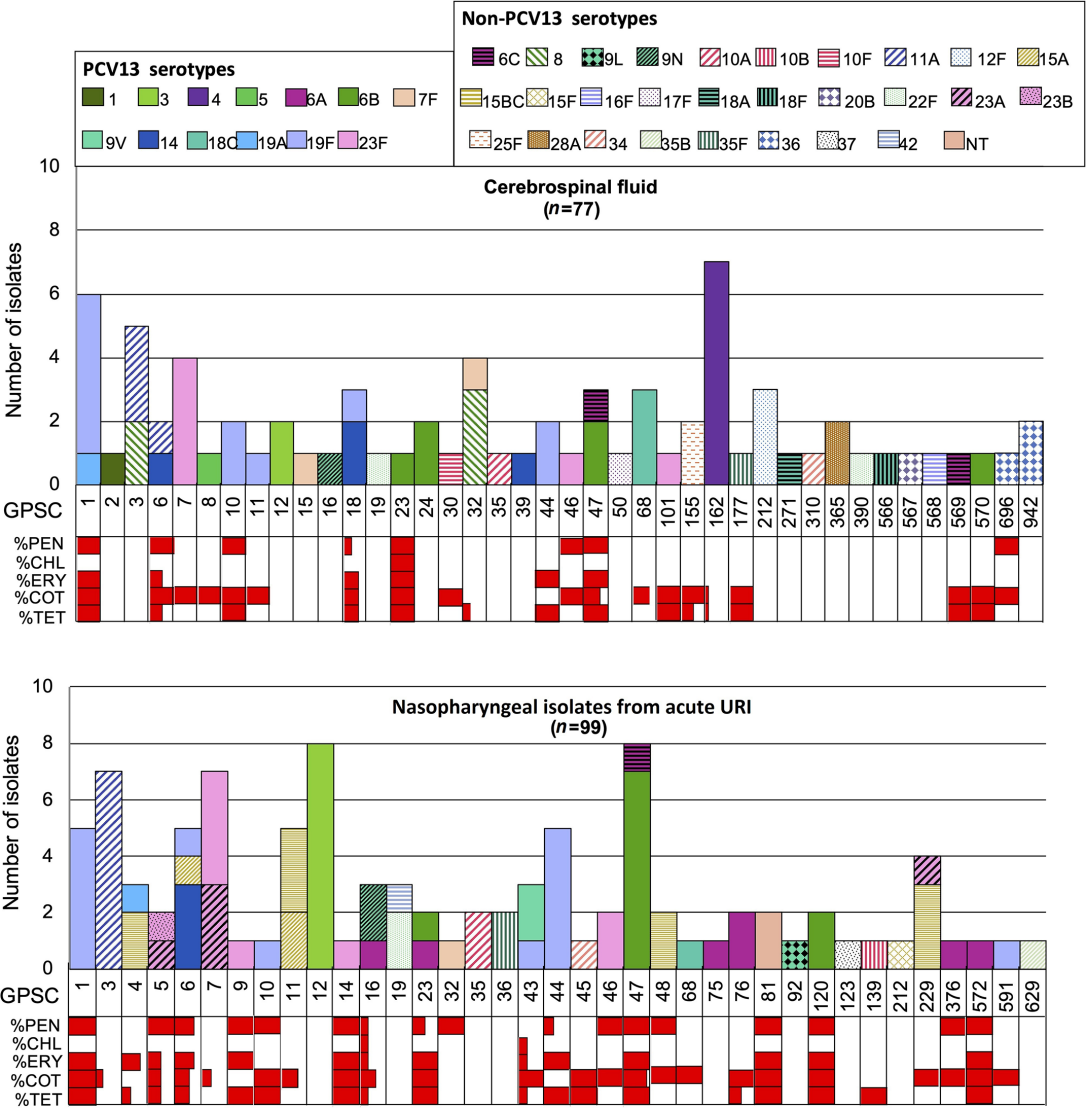
Distribution of global pneumococcal sequence clusters (GPSCs) among *Streptococcus pneumoniae* isolates from cerebrospinal fluid (*n*=77) and nasopharyngeal swabs (*n*=99) from acute URI samples, Russia, 2011–2018. The number of isolates is plotted by GPSC and coloured by serotypes. Vaccine serotypes are represented by solid fill while non-vaccine serotypes are coloured by hatched patterns. The horizontal bars in red indicate the percentage of antibiotic resistance in each lineage. %PEN, penicillin resistance predicted based on the *pbp1a*, *pbp2x*, *pbp2b* sequences; %CHL, chloramphenicol resistance is predicted by the presence of chloramphenicol acetyltransferase gene, *cat*; %ERY, macrolide resistance is predicted by the presence of erythromycin resistance methylase gene *erm*B or macrolide efflux pump gene *mef*A; SXT, cotrimoxazole non-susceptibility was determined by the presence of mutation I100L in *fol*A and/or any indel within amino acid residue 56–67 in *fol*P; TET, tetracycline resistance is predicted by the presence of *tet*M, *tet*(O) or *tet*(S/M) gene without disruption in the promoter region. CSF, cerebrospinal fluid; NPS, nasopharyngeal swab; URI, upper respiratory tract infection.

**Fig. 2.**
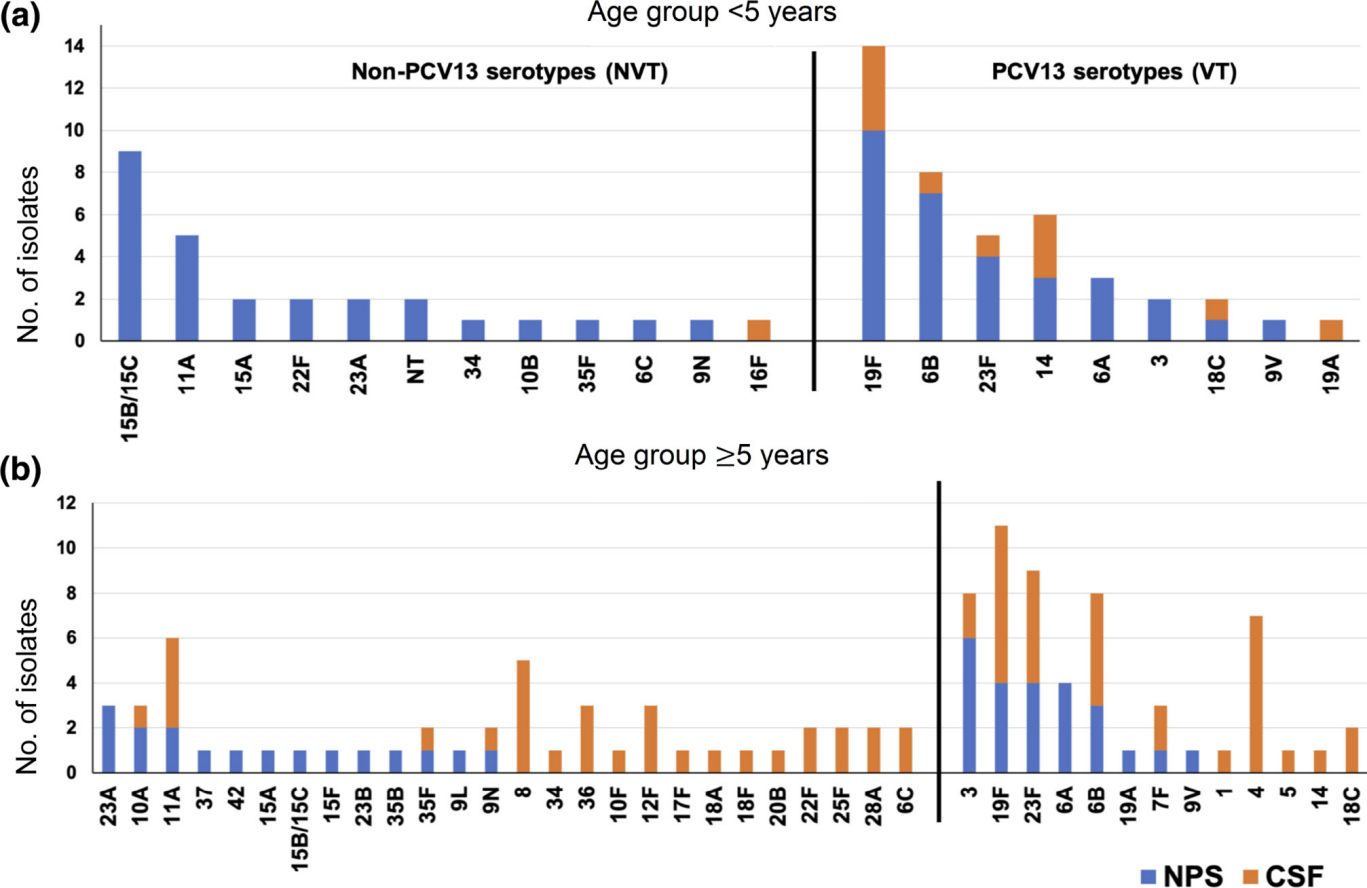
(**a**) Serotype distribution among 12 CSF and 58 nasopharyngeal pneumococcal isolates from children aged <5 years (*n*=70), Russia, 2011–2018. (**b**) Serotype distribution among 65 CSF and 41 nasopharyngeal pneumococcal isolates from individuals aged ≥5 years (*n*=106), Russia, 2011–2018. CSF, cerebrospinal fluid; NPS, nasopharyngeal swab; NT, nontypeable isolates. Three isolates from other non-sterile sites were not included: serotypes 22F, 15B/C and 19F. Vaccine serotypes, PCV13 serotypes; non-vaccine serotypes, non-PCV13 serotypes.

Among the 179 *S *. *pneumoniae* isolates, 42 serotypes were identified, each associated with one to eight pneumococcal lineages (Fig. 1). Serotype 19F was the predominant serotype among both CSF (14.3 %, 11/77) and nasopharyngeal isolates (14.1 %, 14/99) (Fig. 2, Table 2) and one was recovered from other non-invasive disease (33 %, 1/3). The 26 serotype 19F isolates belonged to 8 different lineages: 11 (42 %) belonged to GPSC1, followed by 7 (27 %) GPSC44, 3 (11 %) GPSC10 and a single isolate for each of other 5 GPSCs (GPSC6, GPSC11, GPSC18, GPSC43 and GPSC591). Serotypes 6B (*n*=16) and 23F (*n*=14) were the second and third most prevalent serotypes identified (Table 2). Fifty-six per cent (9/16) of serotype 6B isolates belonged to GPSC47, followed by two isolates in each of GPSC23, GPSC24 and GPSC120 and a single isolate assigned to GPSC570. Over half (57 %, 8/14) of 23F isolates belonged to GPSC7, followed by 21 % (3/14) GPSC46 and a single isolate in each of the GPSCs GPSC14, GPSC101 and GPSC9. The prevalence of both serotypes 4 and 19F was the same at 11 % (7/65) among CSF samples from individuals ≥5 years old. All seven serotype 4 isolates belonged to a single lineage, GPSC162 (CC1222), and were recovered from adults aged between 30 and 57 years old in Moscow from 2011 to 2018.

**Table 2.** The PCV13 coverage and 10 predominant serotypes by age groups and specimen source

	CSF		NPS		Total *n*=176
Serotypes	<5 years (*n*=12)	≥5 years (*n*=65)	<5 years (*n*=58)	≥5 years (*n*=41)	
PCV13 serotypes	11 (91.7 %)	33 (50.8 %)	31 (53.4 %)	24 (58.5 %)	99 (56.3 %)
19F	**4 (33.3 %)** first	**7 (10.8 %)** first	**10 (17.2 %)** first	**4 (9.8 %)** second	25 (14.2 %)
6B	1 (8.3 %)	**5 (7.7 %)** second	**7 (12.1 %)** third	3 (7.3 %)	16 (9.1 %)
23F	1 (8.3 %)	**5 (7.7 %)** second	4 (6.9 %)	**4 (9.8 %)** third	14 (8.0 %)
3	0	2 (3.1 %)	2 (3.4 %)	**6 (14.6 %)** first	10 (5.7 %)
14	**3 (25 %)** second	1 (1.5 %)	3 (5.2 %)	0	7 (4,0 %)
4	0	**7 (10.8 %)** first	0	0	7(4.0 %)
6A	0	0	3 (5.2 %)	**4 (9.8 %)** third	7 (4.0 %)
18C	1 (8.3 %)	2 (3.1 %)	1 (1.7 %)	0	4 (2.3 %)
7F	0	2 (3.1 %)	0	1 (2.4 %)	3 (1.7 %)
19A	1 (8.3 %)	0	0	1 (2.4 %)	2 (1.1 %)
9V	0	0	1 (1.7 %)	1(2.4 %)	2(1.1 %)
Others*	0	2 (3.1 %)	0	0	2 (1.1 %)
Non-PCV13 serotypes	1 (8.3 %)	32 (49.2 %)	27 (46.6 %)	17 (41.5 %)	77 (43.8 %)
11A	0	**4 (6.2 %)** third	**5 (8.6 %)** fourth	2 (4.9 %)	11 (6.3 %)
15B/C	0	0	**9 (15.5 %)** second	1 (2.4 %)	10 (5.7 %)
23A	0	0	2 (3.4 %)	3 (7.3 %)	5 (2.8 %)
22F	0	2 (3.1 %)	2 (3.4 %)	0	4 (2.3 %)
8	0	**5 (7.7 %)** second	0	0	5 (2.8 %)
15A	0	0	2 (3.4 %)	1 (2.4 %)	3 (1.7 %)
nt†	0	0	2 (3.4 %)	0	2 (1.1 %)
12F	0	3 (4.6 %)	0	0	3 (1.7 %)
10A	0	1 (1.5 %)	0	2 (4.9 %)	3 (1.7 %)
35F	0	1 (1.5 %)	1 (1.7 %)	1 (2.4 %)	3 (1.7 %)
6C	0	2 (3.1 %)	1 (1.7 %)	0	3 (1.7 %)
9N	0	1 (1.5 %)	1 (1.7 %)	1 (2.4 %)	3 (1.7 %)
25F	0	2 (3.1 %)	0	0	2 (1.1)%
28A	0	2 (3.1 %)	0	0	2 (1.1 %)
34	0	1 (1.5 %)	1 (1.7 %)	0	2 (1.1 %)
36	0	3(4.6 %)	0	0	3 (1.7 %)
Others‡	1 (8.3 %)	5 (7.7 %)	1 (1.7 %)	6 (14.6 %)	13 (7.4 %)

Other pneumococcal isolates recovered from non-sterile site swabs: two eye swabs (serotypes 22F and 15A) and one vaginal swab (serotype 19F) were not included in the table.

*Other PCV13 serotypes included serotype 1 and 5.

†nt, non-typable isolates.

‡Other non-PCV13 serotypes included 13 serotypes: 23B, 20B, 18F, 18A, 17F, 16F, 15F, 37, 10B, 10F, 9L, 42 and 35B with representation of 1 isolate each.

§The top 10 serotypes with representation of at least 3 isolates are written in bold. The ranking is indicated in superscript.

CSF, cerebrospinal fluid; NPS, nasopharyngeal swab.

Among the 60 GPSCs, there were 25 that only expressed VTs while 28 only expressed NVTs. Seven GPSCs (GPSC4, GPSC6, GPSC7, GPSC11, GPSC16, GPSC32 and GPSC47) expressed both VTs and NVTs (Fig. 1). Among these lineages, GPSC32 expressed serotypes 7F (VT) and 8 (NVT). The latter was only detected in CSF samples, indicating its high invasive disease potential, which was also described in previous studies [25, 41]. In addition, GPSC6 and GPSC47 expressing both VTs and NVTs were observed in CSF and nasopharyngeal samples and exhibited multidrug resistance (Fig. 1).

Serotypes 11A, 15B/C, 23A, 8 and 22F were the five most prevalent NVTs in the collection and each of them was expressed by two to four GPSCs (Fig. 1, Table 2). Both serotypes 8 (GPSC3 and GPSC32) and 11A (GPSC3 and GPSC6) were in the top five serotypes detected in CSF samples from individuals aged 5 years or older. Serotypes 15B/C (GPSC4, GPSC11, GPSC48 and 229) and 11A (GPSC3) were among the top five serotypes among the nasopharyngeal isolates from children aged under 5 years. Serotypes 15B/C and 23A were only observed in nasopharyngeal samples (Fig. 1, Table 2). Among the GPSCs found in the top five NVTs, only GPSC6 was MDR, exhibiting resistance to penicillin, erythromycin, cotrimoxazole and tetracycline.

**Fig. 3.**
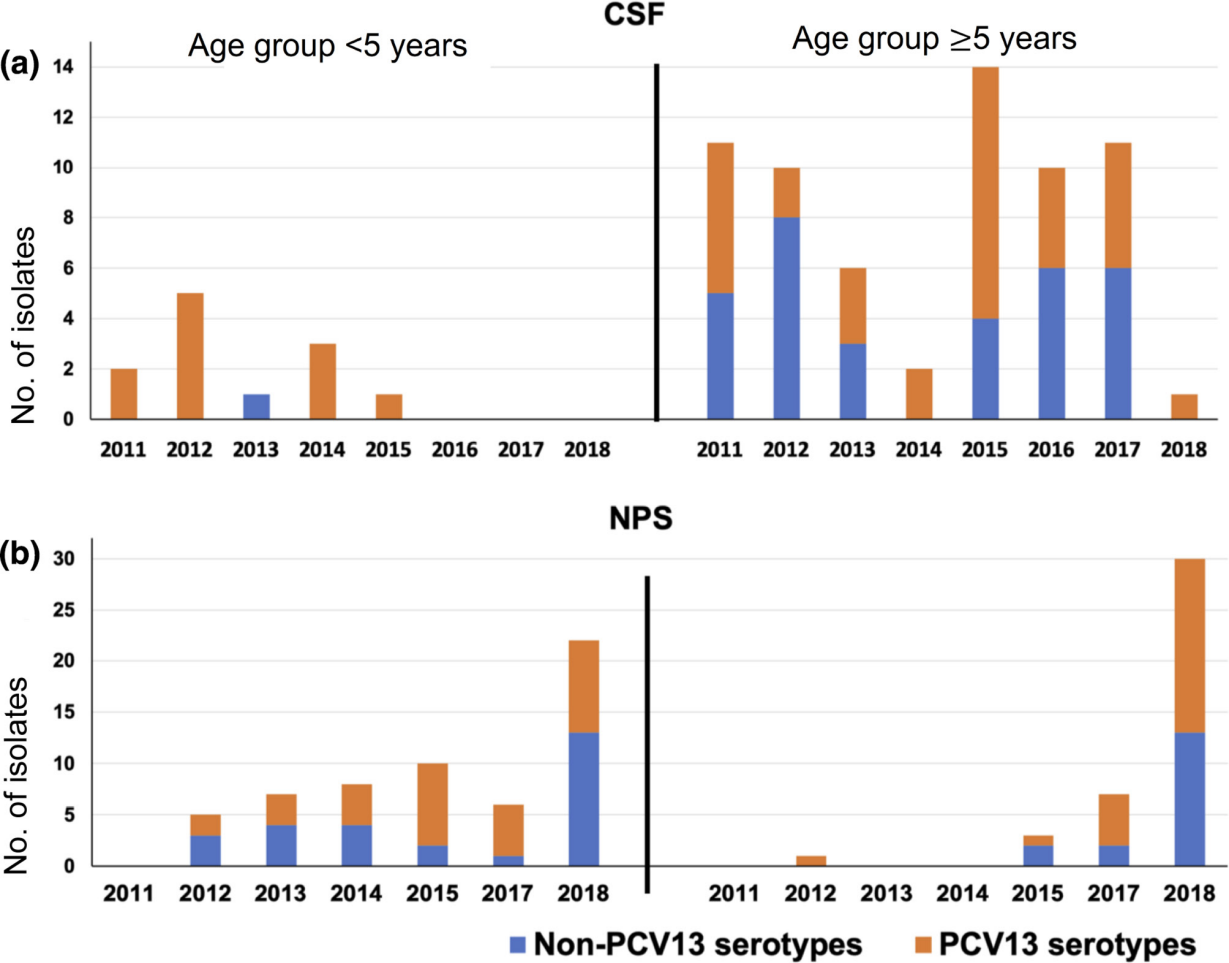
Yearly distribution of PCV13 and non-PCV13 serotypes identified among the pneumococcal isolates obtained from (a) cerebrospinal fluid (CSF) (*n*=77) from patients aged <5 years (*n*=12) and ≥5 years (*n*=65) and (b) nasopharyngeal swabs (NPSs) (*n*=99) from patients aged <5 years (*n*=58) and ≥5 years (*n*=41) between 2011–2018 in Russia. Three isolates from other non-sterile sites were not included: serotypes 22F, 15B/C and 19F.

The yearly distribution of VTs and NVTs among both CSF and nasopharyngeal samples separated by age groups over the study period is shown in Fig. 3. PCV serotype coverage by sample source and age group is listed in Table S5. Notably, 92 % (11/12) of the CSF isolates from children <5 years belonged to PCV13 serotypes; in contrast, only 51 % (33/65) of CSF isolates from individuals aged 5 years and above belonged to PCV13 (*P*=0.0101). In the case of isolates recovered from nasopharyngeal swabs, the PCV13 serotype coverage was 53 and 59 % among individuals <5 and ≥5 of age, respectively. Due to the small number of CSF samples from children aged <5 years old, we could not observe any increase in serotype coverage by higher-valent vaccines. However, increasing serotype coverage was detected among nasopharyngeal isolates collected from children aged under 5 years. PCV13 serotype coverage was 53 % in this group and it increased to 57 % for PCV15 [under review by the US Food and Drug Administration (FDA) and European Medicines Agency (EMA)[42]], 81 % for PCV20 (approved by the FDA)[43] and 83 % for PCV24 (under development). As nasopharyngeal colonization is the prerequisite to causing invasive disease, reducing the carriage of vaccine serotypes that are associated with higher invasive disease potential and carry multidrug resistance in the ecological niche is very likely to reduce IPD cases.

### Capsular switching events

**Fig. 4.**
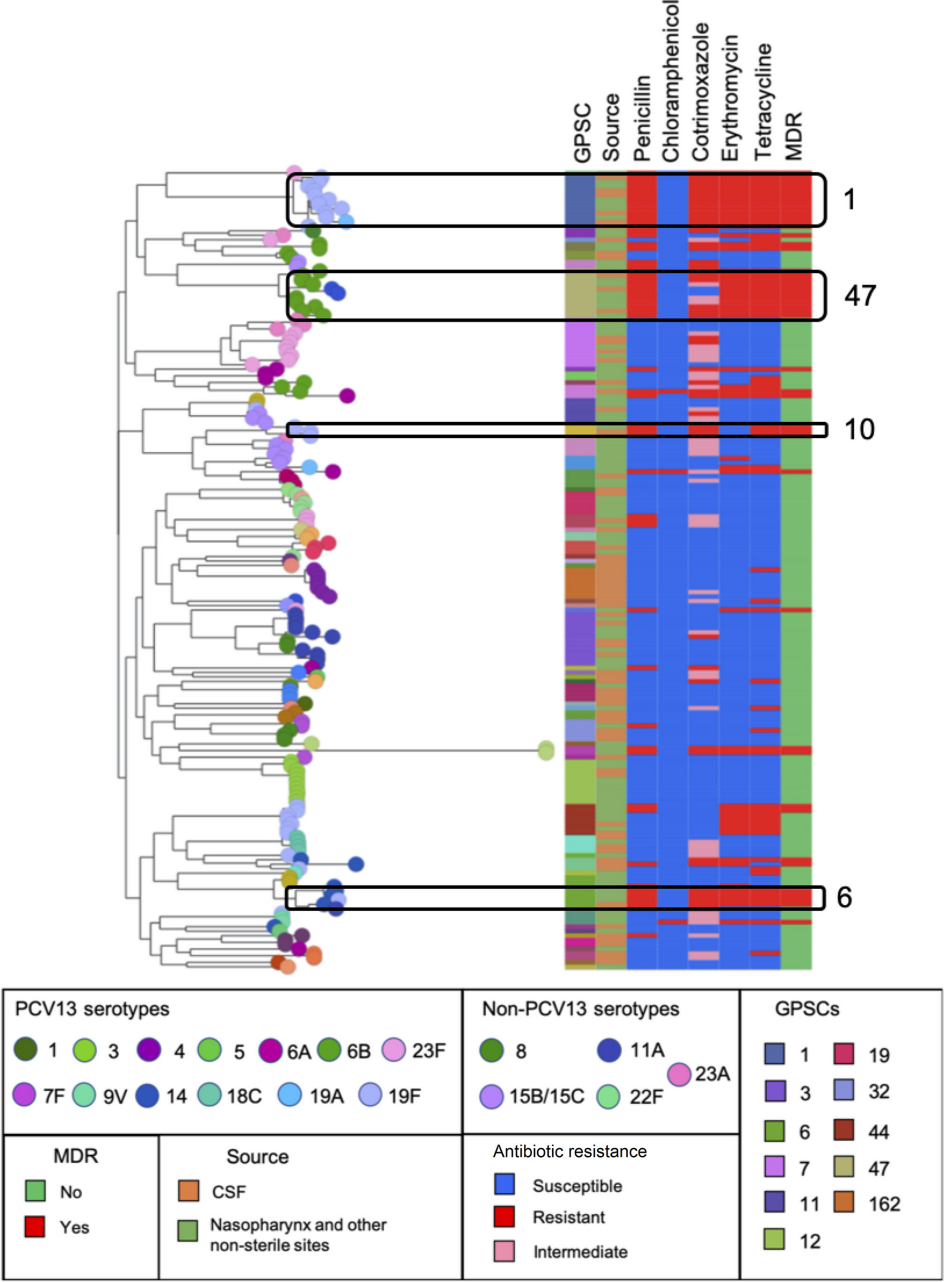
Maximum-likelihood tree of *Streptococcus pneumoniae* isolates from CSF (IPD) (*n*=77), nasopharynx and other non-sterile sites (*n*=102) from Russia, 2011–2018. The tree nodes are coloured by serotypes. The colours of serotypes and GPSCs that are represented by <5 isolates in this collection are not shown in the legend. The clusters highlighted in black boxes represent predominant MDR lineages with ≥3 isolates (GPSC1, 6, 10 and 47). CSF, cerebrospinal fluid; IPD, invasive pneumococcal disease; MDR, multidrug-resistant. Other non-sterile sites, vaginal swab (*n*=1) and eye swabs (*n*=2). This figure can be visualized interactively at https://microreact.org/project/ GPS_Russia.

Change of serotypes (capsular switch) occurs in pneumococcal isolates as a result of mutations or/and recombination in the capsule biosynthesis locus [44]. The whole-genome phylogenetic tree of the 179 isolates revealed high genetic diversity in accordance with GPSCs and observed serotypes (Fig. 4). Further, we identified seven potential capsular-switching events Table S6, of which three were between VTs, two were between NVTs and two were between VTs and NVTs [6B/6C and 19 F/(15B/C)]. None of these potential capsular switches resulted in any change to the penicillin susceptibility of the isolates.

### Antibiotic resistance

Antimicrobial susceptibility testing was performed on a subset of isolates and showed a categorical agreement (susceptible, intermediate and resistant) with the *in silico* resistance prediction at 100 % (19/19) for penicillin, 95.4 % (83/87) for chloramphenicol, 95.34 % (82/86) for erythromycin, 94.25 % (82/87) for cotrimoxazole and 92.6 %(25/27) for tetracycline. The discrepant results are summarized in Table S7 (a–d). Due to the limited phenotypic results and high concordance between phenotype and genotype, we used the *in silico* resistance predictions for further analyses.

Of all 179 isolates, 58.6 % (*n*=105) were predicted as non-susceptible to at least one antibiotic, while 26 % (46/179) were MDR. The prevalence of predicted antimicrobial resistance is summarized in Table 3. Two GPSCs that contributed most to penicillin resistance and multidrug resistance were GPSC1 and GPSC47 (Fig. 4). GPSC1 accounted for 22 (12/55) and 26 % (12/46) of penicillin resistance and multidrug resistance, respectively. Similarly, GPSC47 was responsible for 20 (11/55) and 24 % (11/46) of the penicillin resistance and multidrug resistance cases observed, respectively. All isolates belonging to GPSC1 (*n*=12) and GPSC47 (*n*=11) were VTs, except for two GPSC47 isolates that expressed non-PCV13 serotype 6C. GPSC10 (*n*=3) was another MDR lineage where all the isolates were serotype 19F. Additionally, of eight isolates belonging to GPSC6 four were predicted as MDR and expressed serotypes 14 and 19F (Fig. 4).

**Table 3.** Distribution of predicted antimicrobial resistance of *Streptococcus pneumoniae* isolates from cerebrospinal fluid (*n*=77) and nasopharyngeal swabs (*n*=99) from patients with acute URI and non-invasive diseases (*n*=3) collected in Central and Northwestern Russia, 2011–2018

	No. of resistant isolates (%)					
	CSF		NPS		Non-invasive disease isolates (*n*=3)	
	<5 years (*n*=12)	≥5 years (*n*=65)	<5 years (*n*=58)	≥5 years (*n*=41)		Total (*n*=179)
Penicillin*	5 (42)	12 (18)	25 (43)	12 (29)	1 (33)	55 (31)
Chloramphenicol	0	1 (2)	0	2 (5)	0	3 (2)
Cotrimoxazole†	10 (83)	23 (35)	34 (59)	18 (44)	1 (33)	86 (48)
Erythromycin	4 (33)	11 (17)	24 (41)	12 (29)	1 (33)	52 (29)
Tetracycline	8 (67)	16 (25)	26 (45)	12 (29)	1 (33)	63 (35)
Multidrug resistance‡	6 (50)	9 (14)	20 (34)	10 (24)	1 (33)	46 (26)

The results were interpreted using CLSI guidelines (M100- ED28 : 2018) [29].

Non-invasive disease isolates were recovered from eye swabs (*n*=2) and vaginal swab (*n*=1).

*Penicillin resistance was defined as an MIC of ≥0.12 µg ml^−1^, according to meningitis breakpoints.

†Pneumococcal isolates that exhibited intermediate and full resistance to cotrimoxazole are categorized as resistant isolates.

‡Multidrug resistance was defined as resistance to three or more classes of antibiotics.

CSF, cerebrospinal fluid; NPS, nasopharyngeal swab; URI, upper respiratory tract infection.

There was no significant difference identified in the prevalence of antibiotic resistance between CSF (*n*=77) and nasopharyngeal isolates (*n*=99) from individuals with acute respiratory infection Fig. S5. Among the CSF isolates, VT isolates had a significantly higher prevalence of resistance to penicillin (*P*=0.0 302), erythromycin (*P*=0.0 022), cotrimoxazole (*P*=0.0 022) and tetracycline (*P*=0.0 165) when compared to NVT isolates. They were also significantly associated with multidrug resistance (*P*=0.0 022) Fig. S6A. Higher prevalence of multidrug resistance (*P*=0.0 002) and resistance to penicillin (*P*=0.0 002), erythromycin (*P*<0.0 001), tetracycline (*P*<0.0 001) and cotrimoxazole (*P*=0.0 072) were also observed among nasopharyngeal VT isolates Fig. S6B.

### Antibiotic resistance determinants and pilus types

The genotypes for penicillin resistance showed 83 different patterns of allelic profiles of *pbp1A*, *pbp2B* and *pbp2X*. Seventeen pneumococci with new penicillin-binding protein (PBP) alleles were identified; and among them, 11 isolates belonging to 6 various lineages (GPSC1, GPSC6 GPSC14, GPSC47, GPSC120 and GPSC376) were predicted to be penicillin-resistant, most of them expressing VTs (91 %, 10/11). High genetic diversity was also noted in cotrimoxazole determinant *folA* (7 different amino acid substitution patterns) and *folP* (10 different indel patterns between 56–67th amino acid). Of the 86 pneumococci that were predicted to be non-susceptible to cotrimoxazole, 40 (46.5 %) belonged to the intermediate phenotype (MIC=1–2 µg ml^−1^) and carried mutations in either the *fol*P (*n*=38) or *folA (n=1*) genes or both (*n*=1), whereas 46 (62.7 %) of the isolates belonged to the resistant phenotype (MIC>=4 µg ml^−1^) and carried mutations in both *folA* and f*olP*. Among the 52 pneumococci predicted to be resistant to erythromycin, 49 isolates belonging to diverse genetic backgrounds (GPSCs) were positive for *ermB*, 3 isolates (GPSC1, GPSC4 and GPSC43) for *mefA* and 13 isolates that belonged to GPSC1 (*n*=11), GPSC14 (*n*=1) and GPSC16 (*n*=1) were positive for both. The tetracycline resistance gene *tet*M was found in 63 isolates belonging to 27 different GPSCs. Of 179 isolates, 56 pneumococci (31.3 %) were found to be positive for PI-1 or PI-2 type pili (*rrgA* or *pitB* pilus subunit genes): 38 and 6 of them were predicted to express only PI-1 or PI-2, respectively, and 12 were positive for both. Isolates with pili had a higher prevalence of resistance to penicillin [64 % (36/56) vs 15 % (19/123), *P*<0.0 001], erythromycin [68 % (38/56) vs 11 % (14/123), *P*<0.0 001] and tetracycline [70 % (39/56) vs 20 % (24/123), *P*<0.0 001]. It is notable that all the isolates (*n*=12) that expressed both pilus types belonged to GPSC1 and were MDR.

## Discussion

This study revealed high serotype and lineage diversity among the pneumococcal isolates recovered from meningitis cases and from the nasopharynx of individuals with acute upper respiratory infections in Central and Northwestern Russia. Overall, the VTs 19F, 23F and 6B were predominant, which is consistent with previous studies from Russia [18, 19, 22, 24, 45]. The distribution of dominant pneumococcal serotypes in Russia prior to and shortly after PCV13 introduction in general was similar to the data reported from neighbouring countries in the same period. In Ukraine, serogroup 6 and serotypes 19F, 14 and 23F were most common in both healthy carriers [17, 46] and in meningitis cases in children aged between 6 months to 5 years in 2012–2014 [46]. In Lithuania, serotypes 6B, 19F and 23F were most commonly isolated from children under 6 years of age with acute upper respiratory tract infection in 2012–2013 [47, 48]. In Poland, 91 % of pneumococcal meningitis cases in children <5 years were caused due to PCV13 serotypes in 2008–2011 [49]; subsequently, the number of cases dropped to 64 % in 2012–2015, which might be linked to vaccination of children <5 years with risk factors and local vaccination programmes [49].

Our collection is composed of a mix of globally and regionally spreading lineages. Among them, two pneumococcal lineages [GPSC6 (CC156) and GPSC47 (CC386)] express both VTs and NVTs that cause invasive disease, and being penicillin- and multidrug-resistant, have high potential to undergo serotype replacement and continue to cause pneumococcal infections under vaccine and antibiotic selective pressure.

PCV13 had a serotype coverage of 92 % among CSF isolates from children under 5 years, whereas the serotype coverage was only 51 % for the isolates from individuals aged 5 years and over. In the case of isolates recovered from nasopharyngeal swabs, the PCV13 serotype coverage was 53 and 59 % among individuals <5 and ≥5 years of age. The PCV13 serotype coverage was lower when compared to another study that reported ~70 % PCV13 serotype coverage among nasopharyngeal samples from children aged under 5 years with similar clinical manifestation from Moscow between 2010−2017 [23]. However, our observation was closer to the estimates from a recent multicentre carriage study that reported PCV13 serotype coverage of 59.2 % among the isolates obtained from children aged under 6 years between 2016–2018 from six different cities in Russia [18]. As the collection in the current study was obtained in 2011–2018 from other regions of Russia and in a different healthcare facility in Moscow, the difference in the observed PCV13 serotype coverage could be due to a disparity in vaccine uptake and the time period of sample collection, as well as the relatively small number of isolates in our collection. Future vaccines such as PCV15, PCV20 and PCV24 with greater coverage of the serotypes hold greater promise in reducing the IPD burden.

Increased prevalence of non-vaccine serotypes post-vaccine introduction has been described in multiple studies [50–52]. Similar to the previous studies from Russia, 11A and 15B/C were among the predominant NVTs in the nasopharyngeal isolates [18, 23]. However, the genetic background of the serotype 15B/C isolates was more diverse with four GPSCs: GPSC4 (ST199 and 16296), GPSC11 (ST1262 and 16293), GPSC48 (ST3201 and 3557) and GPSC229 (ST1025), as compared with only two STs (ST1262 and ST1025 grouped in GPSC11 and GPSC229, respectively) observed by Mayanskiy *et al*. [23]. The lineages expressing serotype 15B/C in this study have been detected in different countries. GPSC229 was mainly detected in Europe, while the others were observed across continents [37]. GPSC11 and GPSC48 were listed as the top 10 pneumococcal lineages associated with serotype replacement [53]. In Israel, a significant increase in serotype 15B/C and concurrent decrease in serotype 19A within GPSC11 was observed; this lineage became one of the major lineages causing IPD approximately 3 years after the introduction of PCV13 [53]. As the second most predominant NVT in this study in causing meningitis among individuals ≥5 years old, serotype 8 was also identified as an invasive serotype [25] causing disease in multiple countries, such as Denmark [54], England and Wales [7] (personal communication with Carmen Sheppard and Natalie Groves), South Africa, Israel [53] and Spain [55] in the post-PCV13 period. Most of these serotype 8 isolates are associated with the top globally spreading lineage GPSC3 (CC53/62/100/1012) [25] and to a lesser extent, GPSC32 (CC218/2331).

Our study showed that approximately 26.7 % (16/60) of GPSCs identified were classified as either rare or local pneumococcal lineages, highlighting some regionally specific ones (e.g. GPSC310, 390 and 629) that are under-represented in public and GPS databases and that could be circulating between Central and Northwestern Russia and Eastern European countries (Poland, Latvia, Czech Republic). These lineages were mainly NVTs. Since most of the Eastern European countries have included PCV10/13 in their national immunization programmes, surveillance is needed to detect the emergence of any NVT lineages that are causing invasive disease in a timely manner.

*S. pneumoniae* undergoes frequent capsular switching, independently of vaccine selective pressure [44]. Our analysis echoed similar observations as we identified seven potential capsular switching events that likely occurred both before and after PCV introduction but require further investigation. These potential switches did not change penicillin susceptibility. Two such capsular switching events that might have contributed to vaccine escape were within ST386 (6B/6C) of GPSC47 and ST1262 [19F, (15B/C)] of GPSC11. Frequent capsular switching could generate NVT variants that enable the lineage to survive under vaccine selective pressure, which is a major mechanism for serotype replacement [53].

Our analysis showed that 59 % (105/179) of isolates were predicted to be non-susceptible to at least one antimicrobial and 26 % (46/179) were MDR. *S. pneumoniae* is one of the high-priority pathogens that the WHO suggests monitoring for antimicrobial resistance (AMR) through the Global Antimicrobial Resistance and Use Surveillance System (GLASS) [56]. According to the data available at https://amrmap.net/ (last accessed March 2021) for analysis of antimicrobial resistance data in Russia, among 1255 pneumococcal isolates collected in Russia 2010–2019, 23.1 % were non-susceptible to erythromycin, 2 % to chloramphenicol, 34 % to tetracycline, 38 % to trimethoprim/sulfamethoxazole and 33 % to benzylpenicillin [European Committee on Antimicrobial Susceptibility Testing (EUCAST) clinical breakpoints] [57]. Another study that included non-invasive pneumococci (*n*=863) collected in Moscow 2009–2013 reported that 28 % of isolates were non-susceptible to penicillin, 26 % to erythromycin and 57 % to trimethoprim/sulfamethoxazole (EUCAST clinical breakpoints) [45]. In this study, the isolates were predicted to have a slightly higher prevalence of non-susceptibility to those antimicrobials, with the exception of penicillin (31%) and trimethoprim/sulfamethoxazole (48 %), probably due to the differences in the geographical distribution, specimen sources and thresholds used for interpretation.

Further, our study identified four MDR lineages (GPSC1, GPSC6, GPSC10 and GPSC47). The majority of the isolates (93 %, 28/30) belonging to these four lineages expressed PCV13 serotypes. These four lineages were also found in Belarus and Poland (GPS database, last accessed March 2021), suggesting that the spread of AMR might be linked to the circulation of clones within the Eastern European geographical region. Similar to a previous study [23], VTs were significantly associated with resistance to antimicrobials and with multidrug resistance, suggesting that the implementation of PCV13 is likely to reduce antibiotic-resistant infections by directly decreasing the prevalence of pneumococci with antibiotic resistance, and at the same time, via a secondary effect through a reduction in febrile illnesses that often lead to the use of antibiotics [58].

The main limitation of this study is the small size of the bacterial collection analysed, which hampered our ability to identify possible changes after PCV introduction and over time. It also prevented us from detecting whether any local clonal expansion was occurring in the study region. Another limitation could be non-uniform representation of the samples over the years and also the vaccination rate, which varied across different regions of the country. An additional uncontrolled factor is the possibility that empirical antibiotic treatment prior to sampling might have reduced the culture isolation rate for the pathogen, resulting in altered serotype and lineage distribution within the dataset.

In conclusion, whole-genome sequencing data allowed us to go beyond serotype description to identify pneumococcal lineages that could potentially evade the current PCV, and to place the local findings in a global context. The results suggest that the PCV13 vaccine could be important in reducing invasive disease and antimicrobial resistance. Continued genomic surveillance is needed to reveal the dynamics of the pneumococcal population in Russia in the post-PCV13 period and to generate crucial evidence for continued implementation of current vaccines and to inform next-generation vaccine design.

## Supplementary Data

Supplementary material 1
