# Supplementary material for: Key features of pneumococcal isolates recovered in Central and Northwestern Russia in 2011–2018 determined through whole-genome sequencing
Source: Microb Genom. 2022 Sep 16;8(9):mgen000851. doi: 10.1099/mgen.0.000851 (PMC9676041; doi:10.1099/mgen.0.000851)
Supplement: Supplementary material 1 [file mgen-8-851-s001.pdf]

Supplementary Figures and Tables

Table S1. Number of isolates by age groups, geographical region and specimen source

Specimen source	Age group (years)	Geographical Region		Total
		Moscow	Northwestern and Central Federal Districts	
CSF	<5	12	0	12
	≥5	65	0	65
NPS (acute URI)[a]	<5	43	15	58
	≥5	21	20	41
Other non-sterile site swabs	<5	0	0	0
	≥5	0	3	3
		141	38	179

[a]Nasopharyngeal swabs were collected from individuals with acute upper respiratory tract infection.

CSF, cerebrospinal fluid. NPS, Nasopharyngeal swabs. URI, acute upper respiratory tract infection. Other non-sterile site swabs, eye swab and vaginal swab. Northwestern Federal District - Saint-Petersburg, Leningrad Oblast, Pskov, Petrozavodsk. Central Federal District - Oryol.

Table S2 (full table). GPSCs identified with associated clonal complexes, sequence types and serotypes

GPSC	CC or ST(Related PMEN clone[a])	Number of isolates	Isolated from CSF[b]	Isolated from NPS[c], eye or vaginal swabs	In_Silico_Serotype (n)
1	CC320 (Taiwan[19F]-14/DLV)	12	6	6	**19F(11), 19A(1)**
3	CC62 (Netherlands [8]-33/SLV)	12	1	3	11A(4)
	CC1012		2	4	11A(6)
	CC53 (Netherlands [8]-33/SLV)		2	0	8(2)
7	CC439 (Tennessee[23F]-4/SLV)	11	4	7	**23F(8),** 23A(3)
47	CC386	11	3	8	**6B(9),** 6C(2)
12	CC180 (Netherlands[3-]31/SLV)	10	2	8	**3(10)**
6	CC156 (Spain[9V]-3/SLV)	8	2	6	**19F(1), 14(4),** 15A(2), 11A(1)
44	CC177 (Portugal[19F]-21/SLV)	7	2	5	**19F(7)**
162	CC1222 Poland[6B]-20/DLV)	7	7	0	**4(7)**
11	CC1262	6	1	3	15B/C(3), **19F(1)**
	CC193(Greece[21] -30/DLV)		0	2	15A(2)

19	CC433	5	1	4	22F(4),42(1)
32	CC218 (Denmark[12F]-24/SLV)	5	4	1	**7F(2),** 8(3)
16	CC66 (Tennessee[14]-18/SLV)	4	1	2	9N(3)
	CC81(Spain[23F]-1/SLV)		0	1	**6A(1)**
68	CC102	4	3	1	**18C(4)**
212	CC6202	4	3	1	12F(3), 15F(1)
229	CC1025	4	0	3	15B/C(3)
	ST12511		0	1	23A(1)
4	CC199 (Netherlands[15B] -37/SLV)	3	0	3	**19A(1),** 15B/C(2)
10	CC230 (Denmark[14]-32/SLV)	3	2	1	**19F(2)**
18	CC15 (England[14]-9/SLV)	3	3	0	**14(2), 19F(1)**
23	CC273 (Greece[6B]-22/DLV)	3	1	2	**6B(2), 6A(1)**
35	CC97	3	1	2	10A(3)
43	CC280	3	0	3	**9V(2), 19F(1)**

46	CC30	3	1	2	**23F(3)**
5	CC12742	2	0	1	23B(1)
	CC172 (Columbia ^{23F}-26/SLV)		0	1	23A(1)
24	CC172	2	2	0	**6B(2)**
36	CC1635	2	0	2	35F(2)
48	CC12127	2	0	2	15B/C(2)
76	CC490	2	0	**2**	**6A(2)**
81	CC8991	2	0	2	NT(2)
120	CC1590	2	0	2	**6B(2)**
155	CC105	2	2	0	25F(2)
365	CC225	2	2	0	28A(2)
942	CC16295	2	2	0	36(2)
2	CC615 (USA^{1}-29/SLV)	1	1	0	**1(1)**
8	CC289 (Columbia ^{5}-19/SLV)	1	1	0	**5(1)**

9	CC63 (Sweden[15A]-25/SLV)	1	0	1	**23F(1)**
14	ST3176	1	0	1	**23F(1)**
15	CC191 (Netherlands[7F]-39/SLV)	1	1	0	**7F(1)**
30	CC4084	1	1	0	10F(1)
39	CC124(Netherlands[14]-35/SLV)	1	1	0	**14(1)**
45	CC1439	1	0	1	34(1)
50	ST123	1	1	0	17F(1)
75	ST16297	1	0	1	**6A(1)**
101	CC172	1	1	0	**23F(1)**
123	CC12702	1	0	1	37(1)
139	CC6524	1	0	1	10B(1)
177	CC2991	1	1	0	35F(1)
271	CC1232	1	1	0	18A(1)
310	ST12466	1	1	0	34(1)

376	ST9247	1	0	1	**6A(1)**
390	ST1470	1	1	0	22F(1)
566	CC4651	1	1	0	18F(1)
567	ST7841	1	1	0	20B(1)
568	ST12519	1	1	0	16F(1)
569	ST12482	1	1	0	6C(1)
570	CC273 (Greece^{6B}-22/DLV)	1	1	0	**6B(1)**
572	ST675	1	0	1	**6A(1)**
591	ST16300	1	0	1	**19F(1)**
629	CC4651	1	0	1	35B(1)
696	ST4841	1	1	0	36(1)
972	ST14714	1	0	1	9L(1)
Total	48 CCs and 14 ST singletons	179	77	102	179

[a]PMEN website. Available: http://web1.sph.emory.edu/PMEN/pmen_table2.html.

[b]CSF, cerebrospinal fluid

[c]NPS, NPS, Nasopharyngeal swab.

PCV13 serotypes are highlighted in bold. PMEN, Pneumococcal Molecular Epidemiology Network; SLV - Single Locus Variant; DLV - Double Locus Variant

Table S3: The geographical distribution of seven pneumococcal lineages that were only found in Russia in the Global Pneumococcal Sequencing (GPS) database and their records in pubMLST database

Pneumococcal lineages	ST	GPS database[a] (n)	MLST database[b] (n)	Countries (n)	Serotype (n)[b]
GPSC566	4651	1	1	Russia (1), China (1)	18F (1), NT (1)
GPSC567	7841	1	1	Russia (2)	20B (1), 20(1)
GPSC568	12519	1	0	Russia (1)	16F (1)
GPSC569	12482	1	0	Russia (1)	6C (1)
GPSC570	2779	1	4	Indonesia (1), Russia (2), Kenya (1), Spain (1)	**6B (3), serogroup 6 (1),** 6E[c] (1)
GPSC572	675	1	4	Italy (2), Poland (1), Russia (2)	**6A (4), serogroup 6 (1)**
GPSC591	16300	1	0	Russia(1)	**19F(1)**

[a]The GPS and pubMLST databases were last accessed in March 2021

[b]Number of non-GPS isolates in the MLST database. Vaccine serotypes are written in bold.

[c]6E is a genotype for either serotype 6A or 6B. No further information was provided in the PubIMLST database (last accessed in March 2021)

Table S4: The geographical distribution of some rare lineages found in Russia in the Global Pneumococcal Sequencing (GPS) database and in pubMLST database

Rare lineages	STs	In the study (n)[a]	GPS database (n)	MLST database[b] (n)		Countries (n)[c]
		Russia	Apart from Russia	Russia	Apart from Russia	Apart from Russia
GPSC310	4878/12466/2337/7086	1	7	3	6	**Israel (5), Turkey (2), Latvia (2),Poland (1),** Germany (1), The Gambia (1), South Africa (1)
GPSC390	1470	1	1	1	3	**Poland (2),** The Netherlands (2)
GPSC629	1816/2189	1	1	1	5	**Poland (2), Czech Republic (3),** France (1)

[a]Number of isolates

[b]Non-GPS isolates in the MLST database.

[c]Countries and numbers of isolates in the GPS and pubMLST databases, that were last accessed in April 2019 and in March 2021 respectively. The Eastern European and West Asian countries are in bold.

Table S5. PCV coverage of the circulating *Streptococcus pneumoniae* serotypes in Central and Northwestern Russia, 2011-2018

	No. of CSF isolates (%)		No. of NPS isolates from acute URI (%)		
	<5 yr (n=12)	≥5 yr (n=65)	<5 yr (n=58)	≥5 yr (n=41)	Total (n=176)
PCV10 (GSK)	10 (83)	31 (48)	26 (45)	13 (32)	80 (45)
PCV10 (SII)	10 (83)	22 (34)	28 (48)	18 (44)	78 (44)
PCV13	11 (92)	33 (51)	31 (53)	24 (59)	99 (56)
PCV15[a]	11 (92)	35 (54)	33 (57)	24 (59)	103 (59)
PCV20[a]	11 (92)	48 (74)	47 (81)	29 (71)	135 (77)
PCV24[b]	11 (92)	51 (78)	48 (83)	30 (73)	140 (80)

[a]PCV15 was accepted for priority review for the use in individuals aged ≥18 years old by FDA and PCV20 was approved by the FDA to be used in individuals aged 18 and older (42,43).

[b]PCV24 is still under development.

CSF, cerebrospinal fluid; NPS, nasopharyngeal swabs; URI, upper respiratory tract infection; PCV, pneumococcal conjugate vaccine.

Table S6. Capsular switches in pneumococcal isolates

Isolate	GPSC	CC	ST	Serotype	Vaccine status	Specimen source	Place of isolation	Year of collection
GPS_RU_990	1	320	320	19F	PCV13	NPS	Moscow	2017
GPS_RU_1427	1	320	320	19F	PCV13	NPS	Moscow	2017
GPS_RU_126	1	320	320	19A	PCV13	CSF	Moscow	2014
GPS_RU_2834	6	143	143	14	PCV13	NPS	Moscow	2012
GPS_RU_662	6	143	143	19F	PCV13	NPS	Moscow	2012
GPS_RU_646	11	1262	1262	15B/C	NVT	NPS	Moscow	2012
GPS_RU_1624	11	1262	1262	19F	PCV13	CSF	Moscow	2012
GPS_RU_427	43	280	239	9V	PCV13	NPS	Moscow	2012
GPS_RU_51	43	280	239	19F	PCV13	NPS	Saint-Petersburg	2018
GPS_RU_578	47	386	386	6B	PCV13	NPS	Moscow	2015
GPS_RU_1355	47	386	386	6C	NVT	CSF	Moscow	2016
GPS_RU_423	19	433	433	22F	NVT	NPS	Moscow	2013
GPS_RU_217	19	433	433	42	NVT	NPS	Moscow	2018

GPS_RU_453	212	6202	6202	12F	NVT	NPS	Moscow	2016
GPS_RU_85611	212	6202	6202	15F	NVT	NPS	Moscow	2018

CSF, cerebrospinal fluid; NPS, Nasopharyngeal swab; NVT, non-PCV13 serotype.

Table S7 (A). The discrepancies in results of conventional methods of antibiotic susceptibility testing and WGS prediction of resistance profiles (Chloramphenicol)

Isolate	GPSC	CC	Serotype	Source	WGS prediction	Conventional method[a]	chloramphenicol acetyltransferase gene (*cat*)[b]
GPS_RU_1127	46	CC30	23F	CSF	S	R	negative
GPS_RU_115	3	CC62	11A	NPS	S	R	negative
GPS_RU_571	7	CC439	23F	CSF	S	R	negative
GPS_RU_8301	229	singleton	23A	NPS	S	R	negative

[a]Disc diffusion method.

[b]Chloramphenicol resistance is predicted by the presence of chloramphenicol acetyltransferase gene, *cat.*

NPS, Nasopharyngeal swab; CSF, Cerebrospinal fluid; WGS, Whole genome sequencing; R, resistant; S, susceptible.

Table S7 (B). The discrepancies in results of conventional methods of antibiotic susceptibility testing and WGS prediction of resistance profiles (Cotrimoxazole)

Isolate	GPSC	CC	Serotype	Source	WGS prediction	Conventional method[a]	*folA (I100L)*[b]	*folP(indel)*[b]
GPS_RU_571	7	CC439	23F	CSF	I	R	absent	FOLP_195-ins
GPS_RU_8301	229	singleton	23A	NPS	I	S	absent	FOLP_189-ins
GPS_RU_846	11	CC193	15A	NPS	S	R	absent	absent
GPS_RU_1882	47	CC315	6B	CSF	R	I	absent	FOLP_176-ins
GPS_RU_578	47	CC386	6B	NPS	I	S	absent	FOLP_176-ins

[a]Agar dilutions, E-tests.

[b]Cotrimoxazole non-susceptibility was determined by the presence of mutation I100L in *fol*A and/or any indel within amino acid residue 56-67 in *fol*P.

NPS, Nasopharyngeal swab; CSF, Cerebrospinal fluid; WGS, Whole genome sequencing; R, resistant; S, susceptible.

Table S7 (C). The discrepancies in results of conventional methods of antibiotic susceptibility testing and WGS prediction of resistance profiles (Erythromycin)

Isolate	GPSC	CC	Serotype	Source	WGS prediction	Conventional method[a]	*ermB*[b]	*mefA*[b]
GPS_RU_846	11	CC193	15A	NPS	S	I	negative	negative
GPS_RU_2460	12	CC180	3	NPS	S	R	negative	negative
GPS_RU_2891	47	CC315	6B	NPS	R	S	positive	negative
GPS_RU_1839	572	singleton	6A	NPS	R	S	positive	negative

[a]Disc diffusion method, E-tests.

[b]Macrolide resistance is predicted by the presence of erythromycin resistance methylase gene *erm*B or macrolide efflux pump gene *mef*A.

NPS, Nasopharyngeal swab; CSF, Cerebrospinal fluid; WGS, Whole genome sequencing. R, resistant; S, susceptible; I, intermediate.

Table S7 (D). The discrepancies in results of conventional methods of antibiotic susceptibility testing and WGS prediction of resistance profiles (Tetracycline)

Isolate	GPSC	CC	Serotype	Source	WGS prediction	Conventional method[a]	*tetM*[b]
GPS_RU_1029	3	CC53	8	CSF	S	R	negative
GPS_RU_274	101	CC172	23F	CSF	R	S	positive

[a]Disc diffusion method.

[b]Tetracycline resistance is predicted by the presence of *tet*M, *tet*(O) or *tet*(S/M) gene without disruption in the promoter region.

NP, Nasopharyngeal swab; CSF, Cerebrospinal fluid; WGS, Whole genome sequencing; R, resistant; S, susceptible.

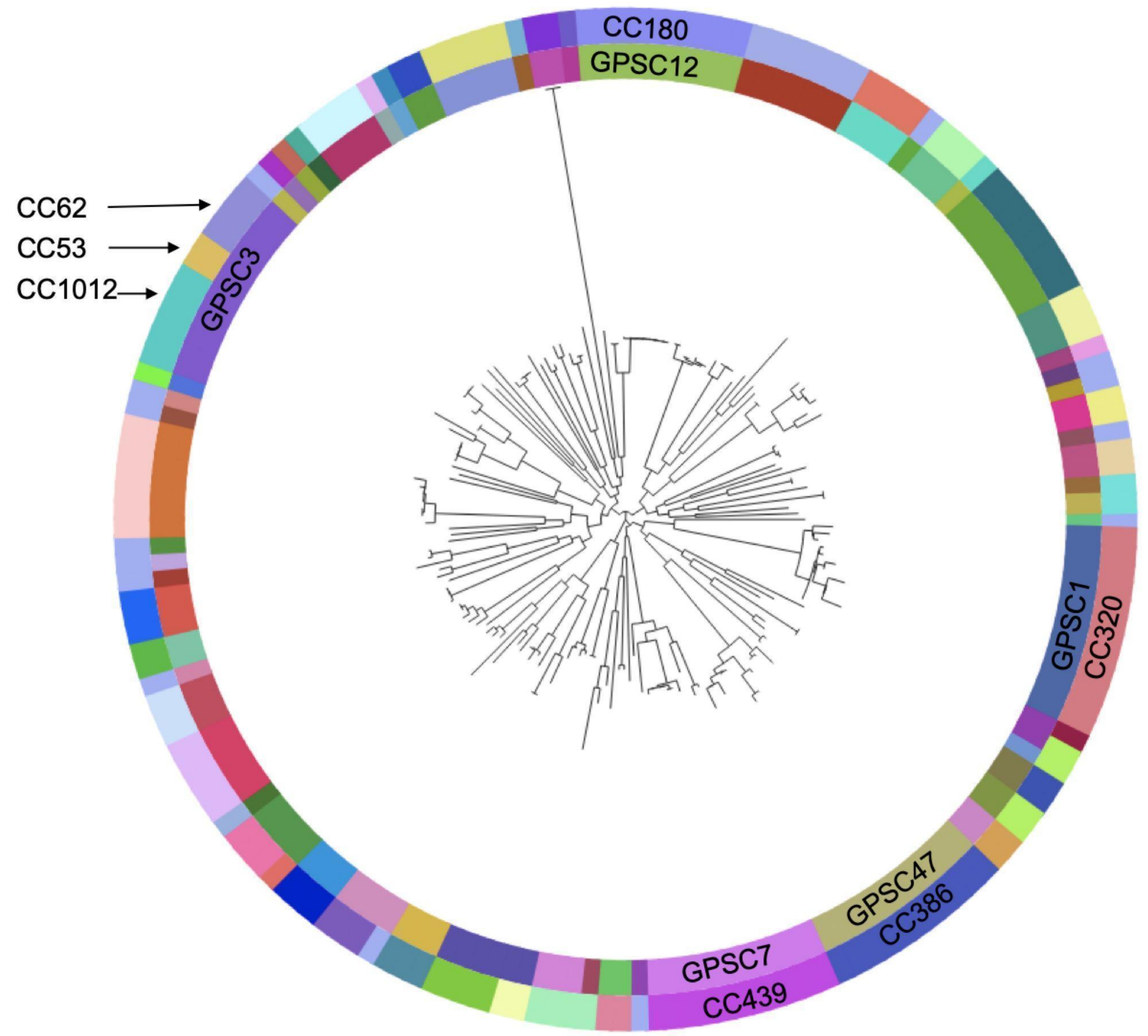

Figure S1: A phylogenetic tree of 179 pneumococcal isolates from Central and Northwestern Russia, 2011-2018. The inner ring shows the Global Pneumococcal Sequence Clusters (GPSCs) and the outer one shows the corresponding clonal complexes (CCs). The five predominant GPSCs and corresponding CCs are labelled. The tree can also be further explored at https://microreact.org/project/GPS_Russia.

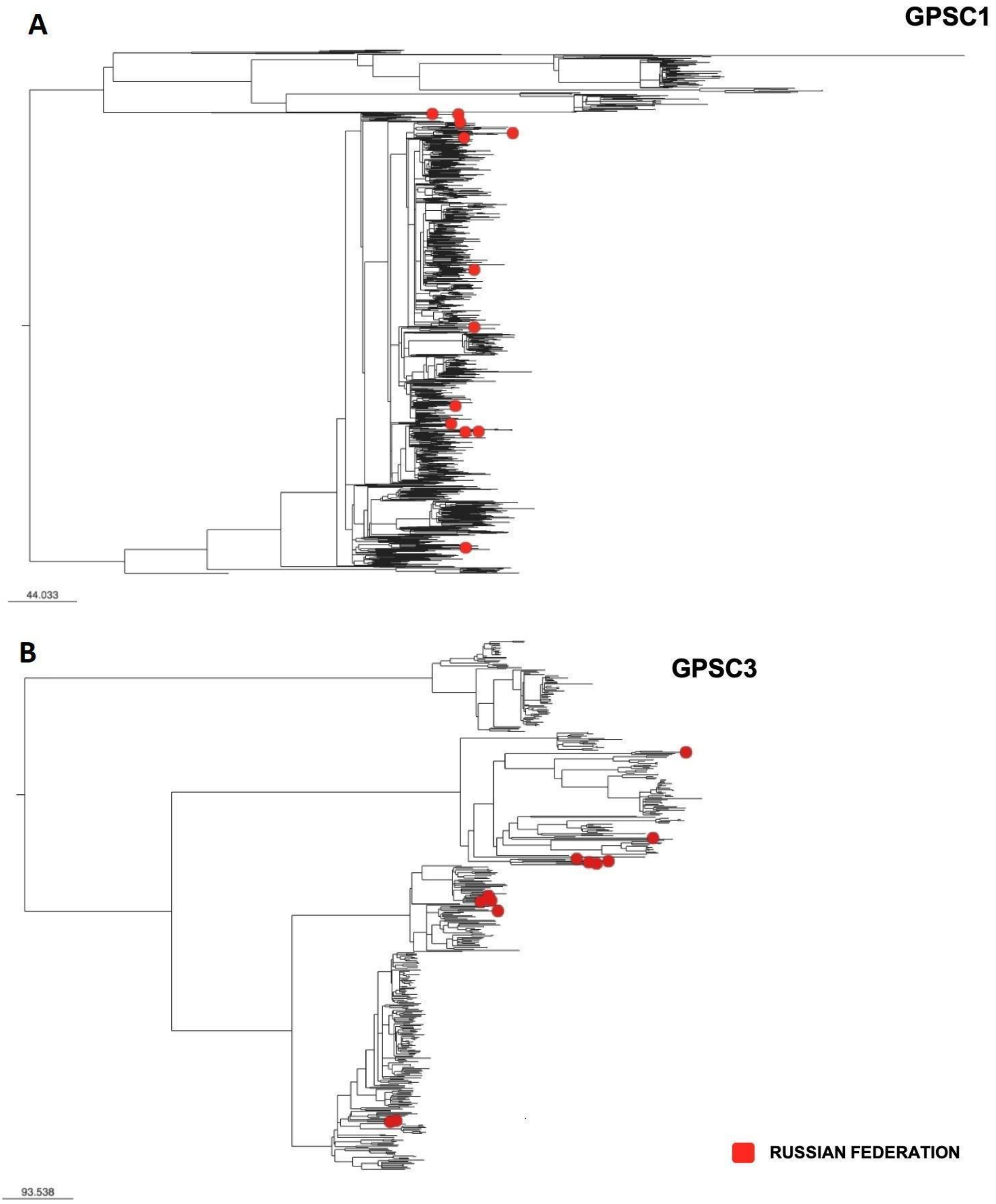

Figure S2. Maximum likelihood trees of pneumococcal lineages or global pneumococcal sequence clusters (GPSCs): A- GPSC1 (n=1149), B- GPSC3 (n=457). GPSC1 includes 12 isolates from Russia. GPSC3 includes 12 isolates from Russia. The tree nodes were colored by country. These figures can be visualized interactively at GPSC1:https://microreact.org/project/3iAgNsS8rzkejRe7nAUgSZ and GPSC3:https://microreact.org/project/kRS1TWgLFD63F1KHpHnNwf

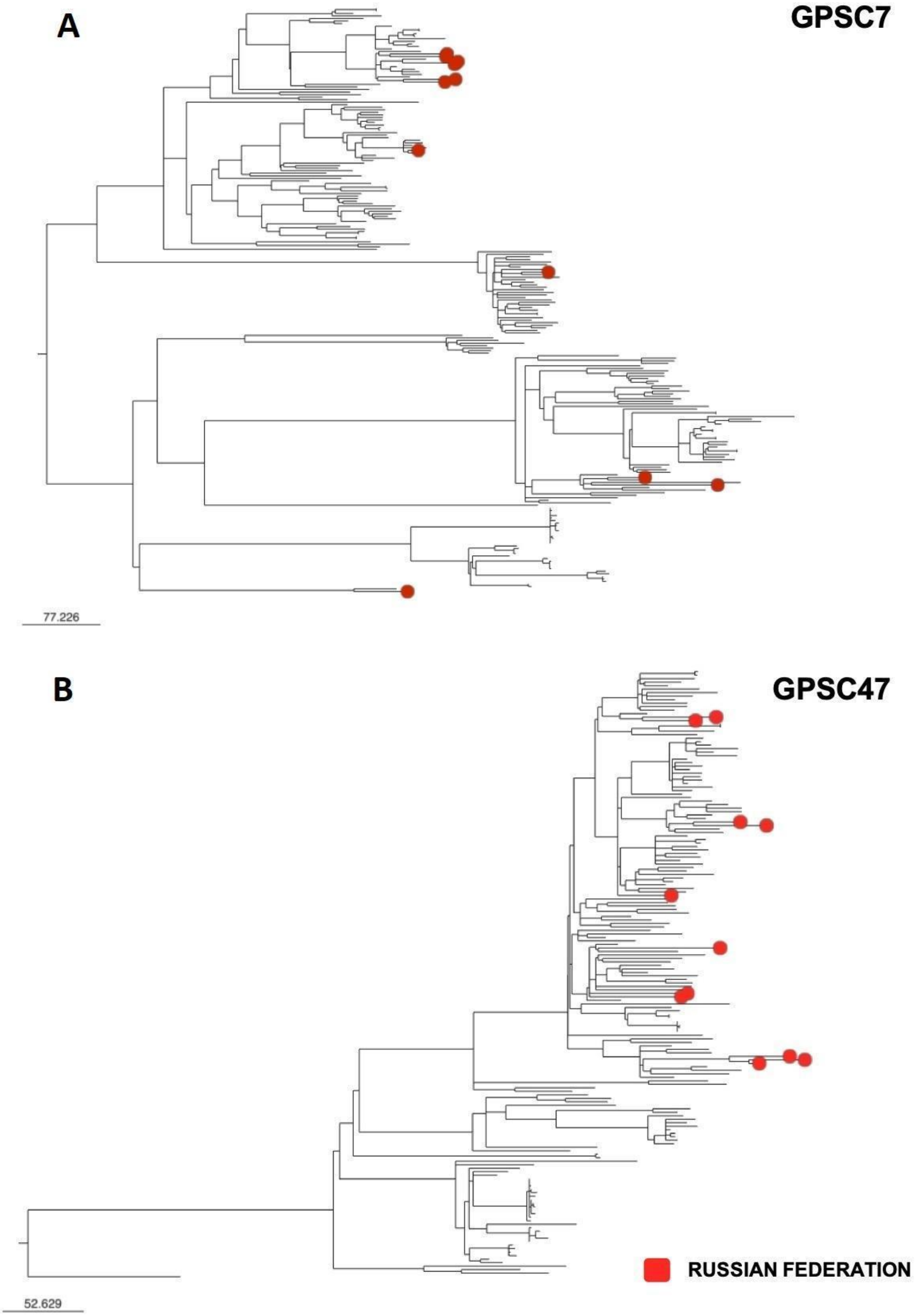

Figure S3. Maximum likelihood trees of pneumococcal lineages or global pneumococcal sequence clusters (GPSCs): A- GPSC7 (n=231), B- GPSC47 (n=174). GPSC7 includes 11 isolates from Russia. GPSC47 includes 11 isolates from Russia. The tree nodes were colored by country. These figures can be visualized interactively at GPSC7:https://microreact.org/project/wpxV17Z2Fhyx8Ds4NudcQq and GPSC47:https://microreact.org/project/rKpFYVmstwSvrwdFTh7CiR

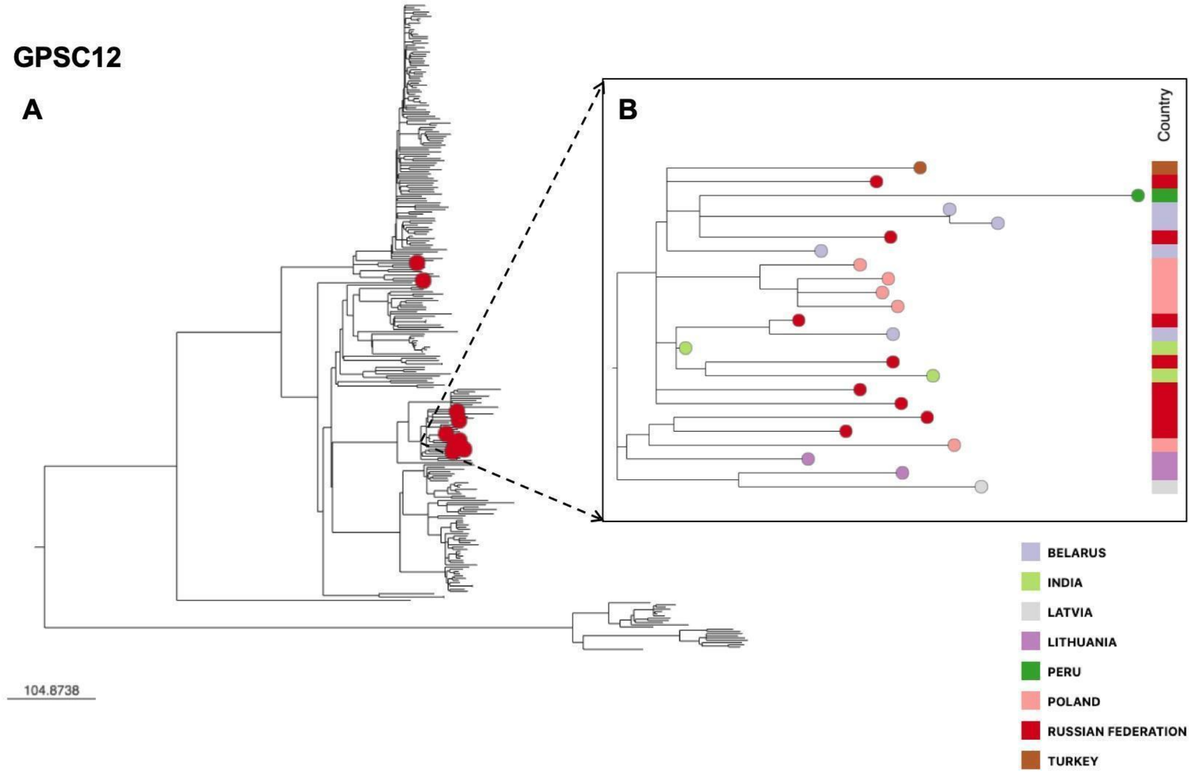

Figure S4. A- Maximum likelihood tree of pneumococcal lineage or global pneumococcal sequence cluster GPSC12 (n=239). GPSC12 includes 10 isolates from Russia. B- Clustering of Russian isolates within GPSC12. The tree nodes were colored by country. This figure can be visualized interactively at GPSC12:https://microreact.org/project/kcbs1VXmgcfJVfZs9W9gYR

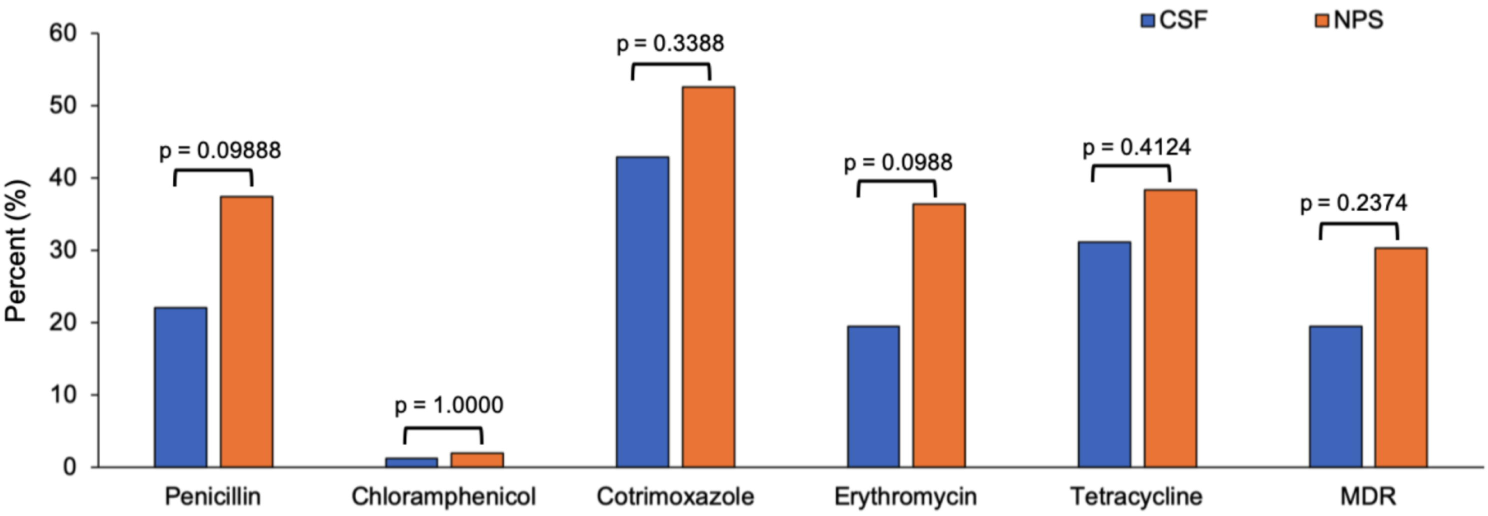

Figure S5. Comparison of the prevalence of antibiotic resistance between cerebrospinal fluid (n=77) and nasopharyngeal (n=99) *Streptococcus pneumoniae* isolates from Russia, 2011-2018. CSF, cerebrospinal fluid; NPS, nasopharyngeal swabs; MDR, multidrug resistance.

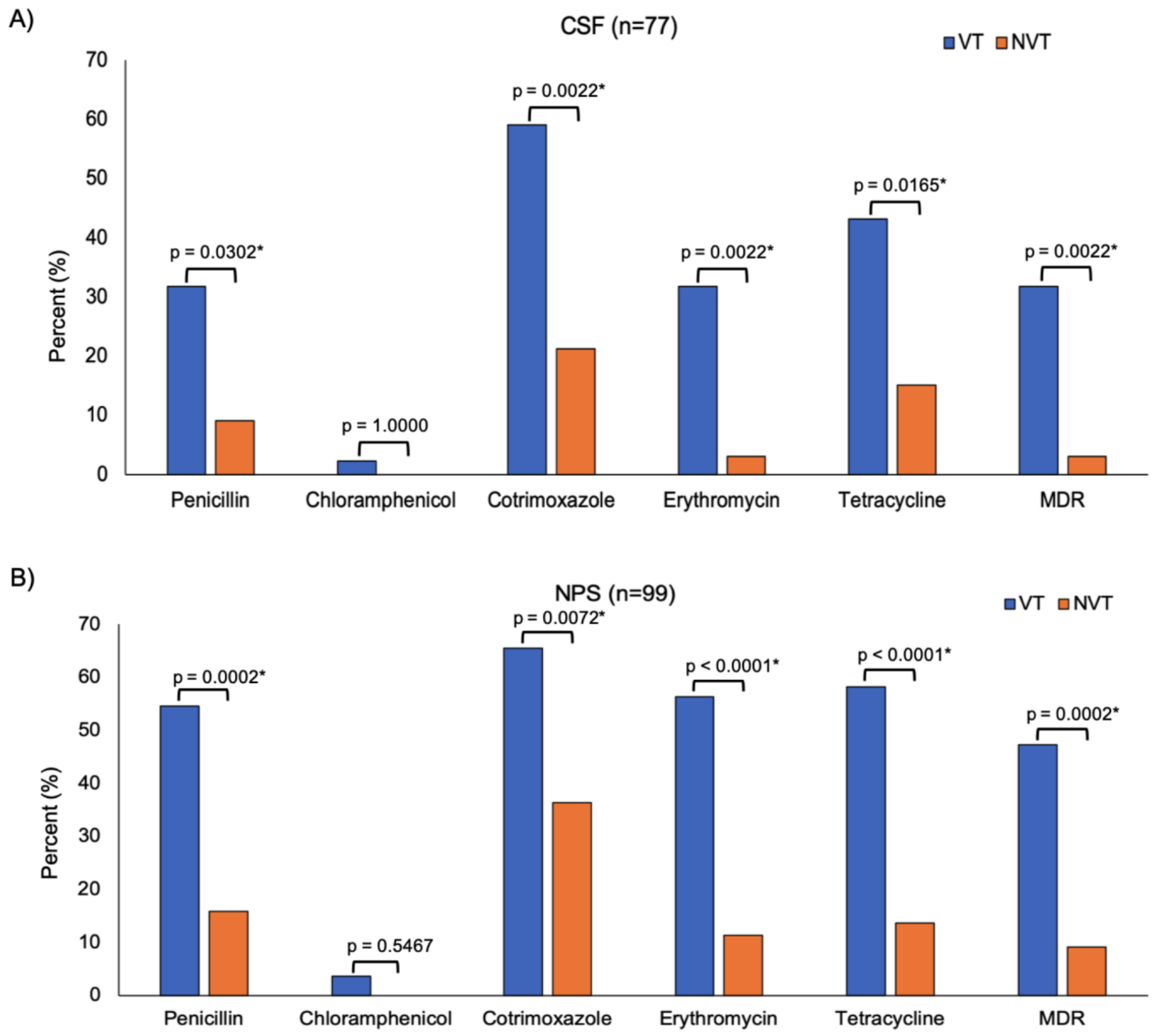

Figure S6. Comparison of the prevalence of antibiotic resistance between PCV13 (including serotype 1, 3, 4, 5, 6A, 6B, 7F, 9V, 14, 18C, 19A, 19F and 23F) and non-PCV13 *Streptococcus pneumoniae* serotypes in (A) CSF (n=77) and (B) NP (n=99) isolates from Russia, 2011-2018. CSF, cerebrospinal fluid; NPS, nasopharyngeal swabs; MDR, multidrug resistance; VT, PCV13 serotypes; NVT, non-PCV13 serotypes.
